# Melatonin Ameliorates Hemorrhagic Transformation via Suppression of ROS-Induced NLRP3 Activation after Cerebral Ischemia in Hyperglycemic Rats

**DOI:** 10.1155/2021/6659282

**Published:** 2021-03-11

**Authors:** Anwen Shao, Shiqi Gao, Haijian Wu, Weilin Xu, Yuanbo Pan, Yuanjian Fang, Xiaoyu Wang, Jianmin Zhang

**Affiliations:** ^1^Department of Neurosurgery, Second Affiliated Hospital, School of Medicine, Zhejiang University, Hangzhou 310009, China; ^2^Collaborative Innovation Center for Brain Science, Zhejiang University, Hangzhou 310009, China; ^3^Brain Research Institute, Zhejiang University, Hangzhou 310009, China

## Abstract

Melatonin is a strong antioxidant which beneficially protects against middle cerebral artery occlusion (MCAO) followed by hemorrhagic transformation in rats; protection includes the reduction of neurological deficits, infarction, and hematoma volume. The molecular mechanisms underlying these neuroprotective effects in the MCAO model have not been clearly identified. This study examined the influence and involved mechanism of melatonin on inflammation in hemorrhagic transformation following hyperglycemia MCAO rat model. Compared with the MCAO group, MCAO+dextrose (DX) group showed worse neurological function and higher infarction and hematoma volume. Interestingly, the protein expression of Nod-like receptor protein 3 (NLRP3) inflammasome increased in the MCAO+DX group compared with the MCAO group, which indicated that NLRP3 inflammasome may be involved in the DX-induced hemorrhagic transformation following MCAO. Then, three dosages of melatonin were intraperitoneally injected 2 h after MCAO induction. Melatonin treatment attenuated inflammatory response by inhibiting the reactive oxygen species (ROS) and NLRP3 inflammasome, alleviating neuronal injury, and reducing infarction and hematoma volume, finally improving neurological score. Melatonin also repressed cortical levels of proinflammatory cytokine IL-1*β*, which were increased 24 h after hyperglycemia MCAO. In order to identify the potential mechanisms, we further revealed that nigericin administration reversed the neuroprotective effect of melatonin by promoting NLRP3 inflammasome activation. In general, this present study reveals that melatonin prevents the occurrence of hyperglycemia-enhanced hemorrhagic transformation, and this effect might be beneficial to attenuate neurological dysfunction via suppressing the inflammatory response after MCAO which possibly associated with the inhibition of the ROS/NLRP3 inflammasome pathway.

## 1. Introduction

Spontaneous hemorrhagic transformation (HT), which is prevalently secondary to ischemic stroke, can be triggered by several pathophysiological changes and the thrombolytic therapy [1]. Clinically, diabetes is an important risk factor to induce HT [2, 3] and, therefore, in animal models, hyperglycemia is used to induce HT after cerebral infarction [4, 5]. The mechanisms of hyperglycemia-enhanced HT, which involves oxidative stress [2, 3, 6], are not completely understood. Moreover, effective drugs or therapeutic strategies available to attenuate hyperglycemia-enhanced HT are very limited either in clinic trials or animal models.

Melatonin (N-acetyl-5-methoxytryptamine) is an amine hormone which mainly synthesized from tryptophan and serotonin metabolism. It is predominantly secreted from the pineal gland and found to play an important role in neuroprotection as an effective antioxidant [7, 8]. Our previous studies have showed that melatonin exerted neuroprotective effect by inhibiting inflammation and apoptosis in early brain injury after subarachnoid hemorrhage, which may associate with the antioxidative effect of melatonin [9]. It has been reported that melatonin has the function to attenuate HT via improving postischemic preservation of the blood-brain barrier permeability [10–12]; however, the mechanism has not been clarified. Oxidative stress is one of predominant inducements during hyperglycemia-enhanced HT [2, 3, 6]; as an antioxidant agent, melatonin has a potential function to inhibit the process of oxidative stress in hyperglycemia-enhanced HT, which may result in ameliorating the symptoms and inflammatory response of HT. Thus, the in-depth study of melatonin and its pathway may provide clinically relevant therapies for hyperglycemia-enhanced HT.

The Nod-like receptor protein 3 (NLRP3, NALP3, cryopyrin) inflammasome is a cytoplasmic complex known to be a key mediator among the innate immune system, which has been widely found to be activated by extensive danger signals that derived from microorganisms and metabolic dysregulation [13]. Under the stimulation of these endogenous and exogenous danger signals, apoptosis-associated speck-like protein containing a caspase recruitment domain (ASC) and procaspase 1 were recruited to NLRP3 eventually forming the NLRP3 inflammasome, which subsequently triggers the activation of caspase 1 (cleaved caspase 1) and secretion of proinflammatory cytokines like IL-1*β* [14]. According to previous studies, NLRP3 inflammasome was showed to be one of the main pathogenic factors during the course of subarachnoid hemorrhage, intracerebral hemorrhage, and cerebral ischemic stroke [15–18]. The activation of NLRP3 inflammasome necessitates an NLRP3-binding protein, thioredoxin-interacting protein (TXNIP), which is released from oxidized thioredoxin (TRX) secondary to high concentrations of reactive oxygen species (ROS) [19]. Subsequently, TXNIP directly binds to a specific region of NLRP3 with abundant leucine which leads to assembly and activation of inflammasome [19]. The inhibitory effect of melatonin on NLRP3 has been reflected in the process of inflammation, apoptosis, and pyroptosis during many diseases, which may be regulated by traditional signaling pathways like Toll-like receptor 4 (TLR4)/nuclear factor kappa B (NF-*κ*B) [20, 21] or microRNA like miR-141 and miR-223 [22, 23]. Nevertheless, the downregulation of NLRP3 by melatonin through ROS scavenging seems to be involved in more researches. ROS can also induce mitochondrial damage to trigger the NLRP3 inflammasome formation; therefore, more recent studies have found that the elimination of NLRP3 may be achieved by the improvement of mitochondrial dysfunction with melatonin treatment [24, 25]. As we can see, ROS is the important intersection between the relationship of melatonin and NLRP3; thus, melatonin may attenuate inflammation induced by NLRP3 inflammasome through inhibiting the ROS/NLRP3 pathway in hyperglycemia-enhanced HT. To verify this hypothesis, nigericin, one of canonical NLRP3 inflammasome agonists, was introduced here to increase the activation of NLRP3 inflammasome and downstream inflammatory response at the condition of melatonin treatment [26]. A recent study in CNS has revealed that microglia were able to form a functional NLRP3 inflammasome and secrete abundant IL-1*β* [27].

In this present study, we hypothesize that melatonin alleviates hyperglycemia-enhanced HT via the ROS/NLRP3 pathway in hyperglycemic MCAO rats. We try to answer two topics in this study: first, whether melatonin can attenuate hyperglycemia-enhanced HT. Second, what is the role of NLRP3 inflammasome in hyperglycemia-enhanced HT, and can melatonin attenuate hyperglycemia-enhanced HT through the ROS/NLRP3 pathway?

## 2. Materials and Methods

We conducted our study mainly through the following three steps. Firstly, we reinvestigated the aggravation on HT and neurological deficit and the expression change on the component of the ROS/NLRP3 pathway by the treatment of dextrose at MCAO rats. Secondly, on the basis of the first step, melatonin was administrated to determine its treatment effect from multiple dimensions in hyperglycemic rats after MCAO. Thirdly, through the administration of nigericin, we try to explicate the mechanism of melatonin's neuroprotection effect in hyperglycemic rats after MCAO.

### 2.1. Animals

All of the procedures were approved by the Institutional Animal Care and Use Committee of Zhejiang University and met the accordance of National Institutes of Health. Health adult male Sprague Dawley rats (8–12 weeks, 250–300 g) were purchased from SLAC Laboratory Animal Co., Ltd. (Shanghai, China). The animals were housed in humidity and temperature-controlled conditions with 12 h day/night cycle and free access to food and water.

### 2.2. Hyperglycemia Induction and MCAO

In order to induce acute hyperglycemia condition, 50% dextrose (6 mL/kg) was intraperitoneally injected to the rats 30 minutes before they were modeled by MCAO. Anesthesia was induced by intraperitoneal injection of 80 mg/kg ketamine and 10 mg/kg xylazine followed by subcutaneous injection of 0.1 mg/kg atropine. During the period of operation and postoperation, rats were placed on a feedback-controlled heating pad to maintain the rectal temperature at 37.0°C. MCAO was modeled as previously reported [28]. In short, after exposing and isolating the right external carotid artery (ECA), a suture with a round tip was inserted from the ECA to the internal carotid artery (ICA). Then, the origin of the middle cerebral artery (MCA) was occluded, and this occlusion was removed in two hours after withdrawing the suture. All the same surgical processes except inserting the suture were performed in sham-operated rats.

### 2.3. Drug Administration

Melatonin (St. Louis, MO, USA) was dissolved in ethanol and diluted with 0.9% saline to obtain solution at the dosage of 150 mg/kg. Vehicle (1% ethanol in 1 mL saline) with similar ethanol content was previously showed no measurable impact on measurement results [11, 15]. Melatonin at three different dosages (15 mg/kg, 50 mg/kg, and 150 mg/kg) and vehicles at a dosage of 5 mL/kg were, respectively, intraperitoneally injected to rats at 2 h after MCAO. A preexperiment was conducted to determine the best dose of melatonin and appropriate time point according to the beneficial effects monitored on mortality following MCAO with melatonin treatment. The sham group and the MCAO+vehicle group were treated by vehicle with equal volumes at the same time.

### 2.4. Neurobehavioral Functions

Neurobehavioral functions were estimated using the modified Garcia test, performed by a blinded investigator 24 hours after MCAO [29]. The total scores conferred to each rat consisted by 7 individual test scores (spontaneous activity, symmetry in the movement of four limbs, forepaw outstretching, climbing, body proprioception, response to vibrissae touch, and beam walking). The scores were graded on a series of scales from 3 (most severe deficit) to 21 (maximum) [30].

### 2.5. 2,3,5-Triphenyltetrazolium Chloride Staining

In order to investigate the infarct volume at 24 hours after MCAO, 2,3,5-triphenyltetrazolium chloride monohydrate staining was performed as described previously [28]. Standard methods (whole contralateral hemisphere volume-nonischemic ipsilateral hemisphere volume) were conducted to correct the possible interference of brain edema with infarct volume. And the infarcted volume was showed by a percentage of the whole contralateral hemisphere [31].

### 2.6. Spectrophotometric Assay of Hemoglobin

Under adequate anesthesia, intravascular blood of rats was removed by complete transcardial perfusion at 24 hours after MCAO. Then, the rats were sacrificed and the brains obtained were rapidly removed and cut into right hemispheres and left hemispheres. After sufficient mincing and homogenization in phosphate-buffered saline (0.1 mol/L), the brains were made into 10% (wt/vol) homogenates. The volume of cerebral hemorrhage was determined with a previously described spectrophotometric assay by comparing samples with a standard curve obtained from a series of “virtual” models of hemorrhage [5, 28]. The hemoglobin content of the whole hemisphere was expressed as *μ*L of blood per hemisphere.

### 2.7. Measurement of ROS

Cortical samples for measurement were obtained from the basal ganglia on the injured (left) side of sacrificed rats at 24 h after MCAO. The accumulation of intracellular ROS was measured with the ROS/RNS assay kit (Cell Biolabs, Inc., USA) according to the manufacturer's protocols. The observation fluorescence of ROS was read at 485 nm with a microplate reader, and the content of ROS in every experimental group was calculated from the control group whose content of ROS was regarded as normal.

### 2.8. Western Blotting

The brain samples were obtained from the cortex of obstructed hemisphere at 24 hours after MCAO. After homogenization and centrifugation, samples containing target protein were separated by Tris-glycine SDS-PAGE and then transferred to PVDF membranes as previously described [32]. Primary antibodies used were NLRP3 (1 : 200), IL-1*β* (1 : 2000), and cleaved caspase 1 (1 : 200). After being washed briefly three times with TBST, PVDF membranes were incubated for one hour at room temperature with corresponding second antibody.

### 2.9. Immunofluorescence

In order to determine microglial (Iba-1) specific colocalization of NLRP3 in ipsilateral hemisphere after hemorrhagic transformation, we conducted fluorescence staining as described [33]. Briefly, primary antibodies were rabbit anti-NLRP3 polyclonal antibody (MBS175714, 1 : 500, MyBioSource) and goat anti Iba-1 polyclonal antibody (ab5076, 1 : 200; Abcam, Cambridge, MA). After blocking with donkey serum 5% for 2 hours at room temperature (RT) and incubation at 4°C overnight, the sections were treated with donkey secondary antibodies (1 : 200; RT for 2 h) raised against rabbit and goat IgG, conjugated with Texas Red and fluorescein isothiocyanate (Santa Cruz Biotechnology).

### 2.10. Nissl Staining

Nissl staining was performed in this study to evaluate the damage and specific morphology of neurons. After being immersed in cresol violet at 37°C for 20 minutes, coronal sections were successively rinsed, dehydrated, and observed with a light microscope. Neurons with round and palely stained nuclei and typical neuronal morphology were regarded as survival cells when they were sampled [34].

### 2.11. Statistical Analysis

Data were expressed as the mean ± sem. Statistical differences among groups were analyzed by using analysis of variance followed by the Tukey test. The correlation of infarction volume and hemorrhage volume was analyzed using the Pearson correlation coefficient; a *p* value of less than 0.05 was considered statistically significant.

## 3. Results

### 3.1. Hyperglycemia Aggravated Infarction and HT and Increased the Level of ROS and NLRP3 Inflammasome-Related Proteins in MCAO Rats

**Figure 1:**
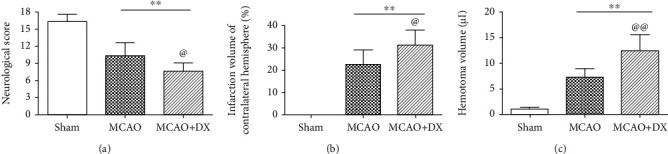
Hyperglycemia exacerbated neurological deficits and aggravated infarction and HT at 24 hours after MCAO: (a) neurologic scores evaluated by the modified Garcia test at 24 hours after MCAO, (b) infarction volume of contralateral hemisphere, and (c) hematoma volume determined by spectrophotometric assay of hemoglobin at 24 hours after MCAO. ^∗∗^*p* < 0.01 vs. the sham group; ^@^*p* < 0.05 vs. the MCAO group; ^@@^*p* < 0.01 vs. the MCAO group. HT: hematoma transformation; MCAO: middle cerebral artery occlusion; DX: dextrose.

**Figure 2:**
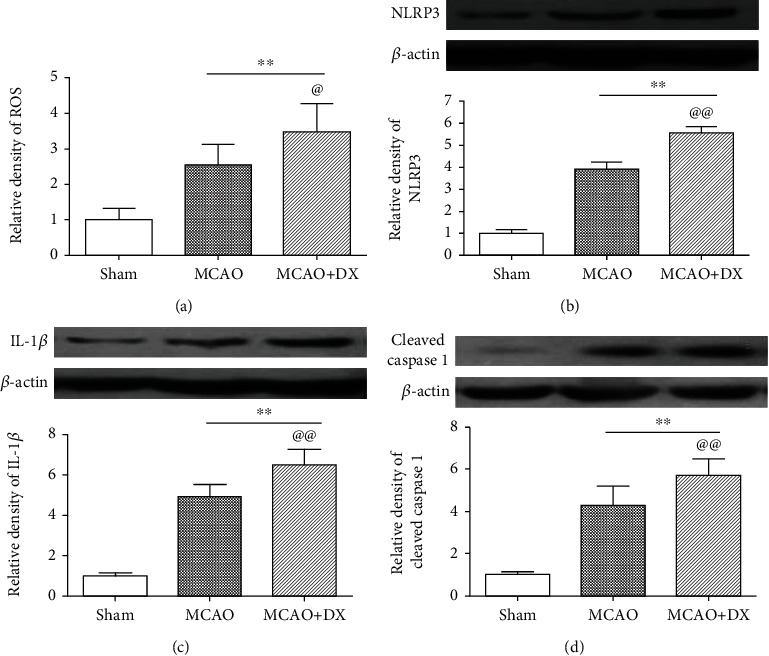
Hyperglycemia increased the level of ROS and the protein expression of NLRP3 inflammasome. (a) Relative density of ROS in the ipsilateral cortex at 24 hours after MCAO. Representative western blot bands and densitometric quantification of NLRP3 (b), IL-1*β* (c), and cleaved caspase 1 (d) in the ipsilateral hemisphere at 24 hours after MCAO. ^∗∗^*p* < 0.01 vs. the sham group; ^@^*p* < 0.05 vs. the MCAO group; ^@@^*p* < 0.01 vs. the MCAO group. NLRP3: nod-like receptor protein 3; MCAO: middle cerebral artery occlusion; DX: dextrose.

Twenty-four hours after MCAO, remarkable reduction in neurological score (Figure 1(a), *p* < 0.01 vs. sham) and serious cerebral infarction followed by HT (Figures 1(b) and 1(c), *p* < 0.01 vs. sham) were observed in operative rats. Meanwhile, the level of ROS (Figure 2(a), *p* < 0.01) and the protein expression of NLRP3 inflammasome including NLRP3, IL-1*β*, and cleaved caspase 1 (Figures 2(b)–2(d), *p* < 0.01) were significantly increased in the ipsilateral cortex when compared to the sham group.

Acute hyperglycemia was induced by intraperitoneal injection of 50% dextrose 30 minutes before MCAO. Hyperglycemia caused obvious neurological deficits (Figure 1(a), *p* < 0.05 vs. MCAO) and significantly aggravated infarction volume (Figure 1(b), *p* < 0.05 vs. MCAO) and hematoma volume (Figure 1(c), *p* < 0.01 vs. MCAO) 24 hours after MCAO. In the MCAO+DX group, the level of ROS increased further compared with the MCAO group (Figure 2(a), *p* < 0.05). Consistently, the expression of NLRP3, IL-1*β*, and cleaved caspase 1 in the MCAO+DX group significantly increased when compared with that in the MCAO group 24 hours after MCAO (Figures 2(b)–2(d), *p* < 0.01).

### 3.2. Pretreatment of Melatonin Alleviated Infarction Volume and Hemorrhagic Transformation and Ameliorated Neurological Deficit in Hyperglycemic Rats 24 Hours after MCAO

**Figure 3:**
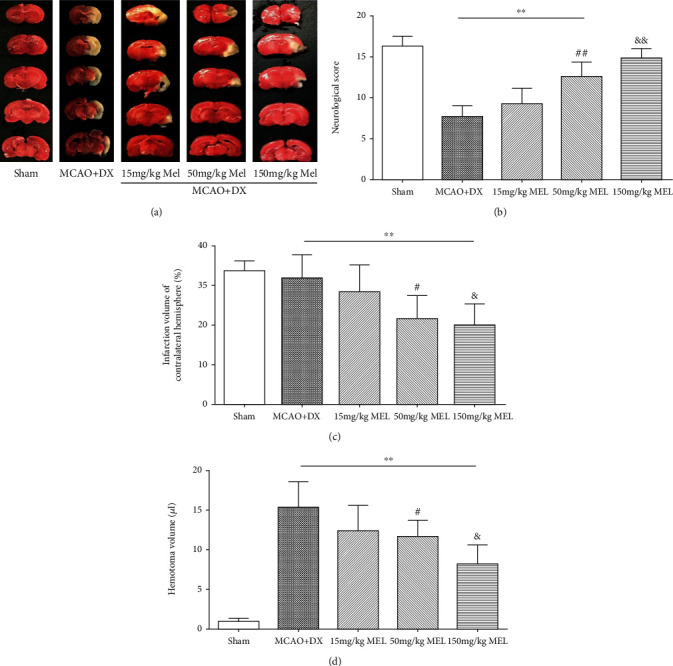
Melatonin alleviated infarction and HT and improved neurological scores in hyperglycemic rats 24 hours after MCAO: (a) representative photographs of whole brain and slices with 2,3,5-triphenyltetrazolium chloride staining at 24 hours after MCAO, (b) neurologic scores evaluated by the modified Garcia test at 24 hours after MCAO, (c) infarction volume of ipsilateral hemisphere, and (d) hematoma volume determined by spectrophotometric assay of hemoglobin at 24 hours after MCAO. ^∗∗^*p* < 0.01 vs. the sham group; ^#^*p* < 0.05 vs. the MCAO+DX group; ^&^*p* < 0.05 vs. the MCAO+DX group. HT: hematoma transformation; MCAO: middle cerebral artery occlusion; DX: dextrose; MEL: melatonin.

Animals were, respectively, treated with melatonin at three different doses (15 mg/kg, 50 mg/kg, and 150 mg/kg) 1 hour before MCAO. Compared with the untreated hyperglycemic MCAO rats, the infarction volume and hemorrhage volume in the MCAO+DX+melatonin group were significantly decreased at a dosage of 150 mg/kg (Figures 3(a), 3(c), and 3(d), *p* <0.05 vs. vehicle). At the treatment of 50 mg/kg melatonin, the infarction volume was similar to the 150 mg/kg melatonin group (Figures 3(a) and 3(c)), while no significant difference of hematoma volume was notified when compared with the vehicle group (Figure 3(d)).

Compared with hyperglycemic MCAO rats without treatment, neurological functions evaluated by neurological scores in the treatment of melatonin at both dosages of 50 mg/kg and 150 mg/kg were significantly improved 24 hours after MCAO (Figure 3(b), *p* < 0.01 vs. vehicle). Meanwhile, no remarkable difference of neurological score, infarction volume, or hemorrhage volume between the MCAO+DX+melatonin group and the MCAO+DX group was observed at a dosage of 15 mg/kg (Figures 3(a)–3(d)).

### 3.3. Pretreatment of Melatonin Reduced the Inflammatory Response and Neuronal Degeneration in Hyperglycemic Rats after MCAO

**Figure 4:**
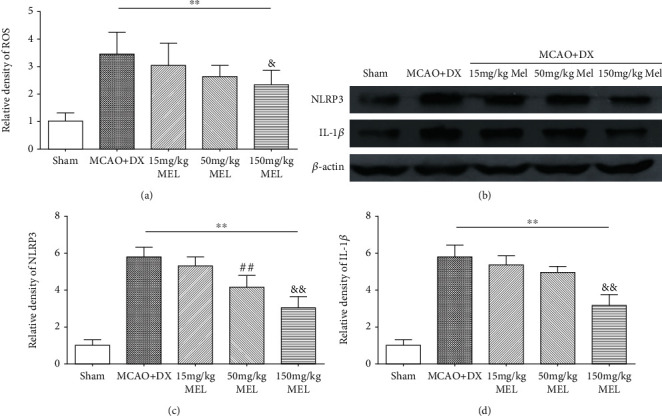
Melatonin reduced the level of ROS, NLRP3, and IL-1*β* in hyperglycemic rats 24 hours after MCAO. (a) Relative density of ROS at 24 hours after MCAO. (b) Representative western blot bands of NLRP3 and IL-1*β*. Densitometric quantification of NLRP3 (c) and IL-1*β* (d) at 24 hours after MCAO. ^∗∗^*p* < 0.01 vs. the sham group; ^#^*p* < 0.05 vs. the MCAO+DX group; ^&^*p* < 0.05 vs. the MCAO+DX group.^##^*p* < 0.01 vs. the MCAO+DX group; ^&&^*p* < 0.01 vs. the MCAO+DX group. MCAO: middle cerebral artery occlusion; DX: dextrose; MEL: melatonin.

There was a significant reduction in the level of ROS in the MCAO+DX+150 mg/kg MEL group compared to the MCAO+DX group 24 hours after MCAO (Figure 4(a), *p* < 0.05 vs. MCAO+DX). Consistently, the protein expression of NLRP3 and IL-1*β* was also significantly decreased at the treatment of 150 mg/kg melatonin compared to the hyperglycemic MCAO rats without treatment (Figures 4(b)–4(d), *p* < 0.05 vs. MCAO+DX). Treatment of 50 mg/kg melatonin could obviously decrease the expression of NLRP3 (Figures 4(b) and 4(c), *p* < 0.05 vs. MCAO+DX) but showed no remarkable effect on the content of ROS and IL-1*β* when compared with the untreated hyperglycemic MCAO rats (Figures 4(a), 4(b), and 4(d)). Similar to the previous result, no obvious level change was observed in the low-dose group when compared with the MCAO+DX group 24 hours after MCAO (Figures 4(a)–4(d)). According to the results mentioned above, the optimum dosage of melatonin was determined at 150 mg/kg in the following experiments.

**Figure 5:**
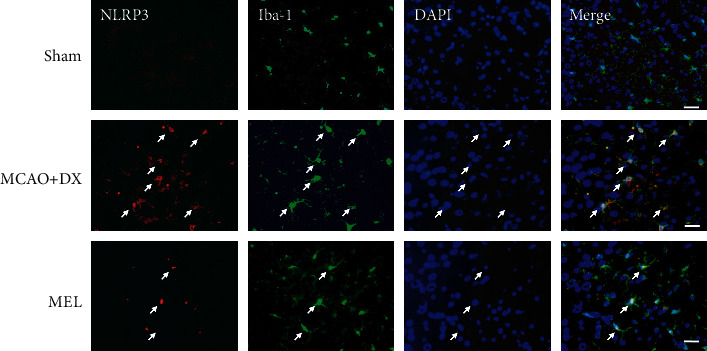
Melatonin reduced the expression of NLRP3 in hyperglycemic rats 24 hours after MCAO. Representative photographs of immunofluorescence staining for NLRP3 (red) expression in microglia (Iba-1, green) in the ipsilateral basal cortex at 24 hours after MCAO. Scale bar = 50 *μ*m. MCAO: middle cerebral artery occlusion; DX: dextrose; MEL: melatonin.

**Figure 6:**
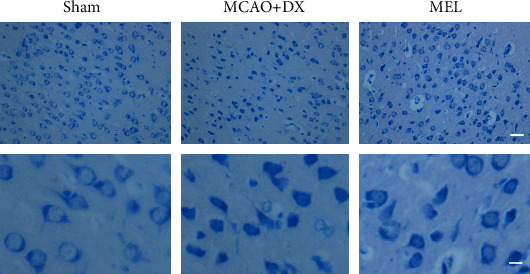
Melatonin alleviated neuronal degeneration in hyperglycemic rats 24 hours after MCAO. Representative micrographs of Nissl staining. Arrows indicated the normal neurons at 24 hours after MCA. MCAO: middle cerebral artery occlusion; DX: dextrose; MEL: melatonin.

With melatonin pretreatment, consistently, the immunofluorescence results also showed the decreased expression of NLRP3 in microglia in hyperglycemic rats after MCAO (Figure 5). A medium-term assessment presented by Nissl staining was performed at 7 days after MCAO. Similar defects were showed by more loss and shrinkage of neurons in the MCAO+DX group compared with the sham group. Moreover, neuronal damage was also remarkably reversed by the treatment of melatonin when compared to the MCAO+DX group (Figure 6).

### 3.4. Nigericin Reversed the Protective Effects of Melatonin on Hyperglycemia-Enhanced Inflammatory Response, Hemorrhagic Transformation, and Neurobehavioral Outcomes

**Figure 7:**
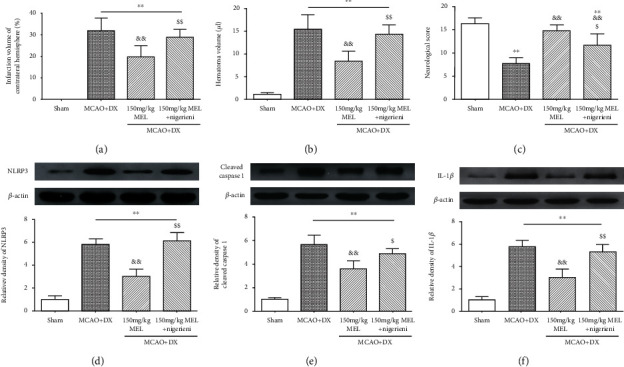
Nigericin reversed the protective effects of melatonin on hyperglycemia-enhanced inflammatory response, hemorrhagic transformation, and neurobehavioral outcomes. (a) Infarction volume of contralateral hemisphere and (b) hematoma volume at 24 hours after MCAO. (c) Neurologic scores evaluated by the modified Garcia test at 24 hours after MCAO. Representative western blot bands and densitometric quantification of NLRP3 (d), cleaved caspase 1 (e), and IL-1*β* (f) in the ipsilateral hemisphere at 24 hours after MCAO. ^∗∗^*p* < 0.01 vs. the sham group; ^&&^*p* < 0.01 vs. the MCAO+DX group; ^$^*p* < 0.05 vs. the MCAO+DX+MEL group; ^$$^*p* < 0.01 vs. the MCAO+DX+MEL group. MCAO: middle cerebral artery occlusion; DX: dextrose; MEL: melatonin.

The i.c.v injection of nigericin at 2 h before MCAO significantly reincreased the level of NLRP3 in hyperglycemic MCAO rats at the treatment of melatonin, demonstrating the NLRP3 inflammasome activation efficacy of nigericin in the present research (Figure 7(d), *p* < 0.01 vs. MCAO+DX+MEL). Moreover, NLRP3 inflammasome activation by nigericin pretreatment significantly increased the downstream protein expression including cleaved caspase 1 and IL-1*β* in the MCAO+DX+MEL+nigericin group compared with that in the MCAO+DX+MEL group at 24 hours after MCAO (Figures 7(e) and 7(f)).

Consistently, significant increased infarction volume and hematoma volume were observed at 24 hours after MCAO in the MCAO+DX+MEL+nigericin group compared to the MCAO+DX+MEL group (Figures 7(a) and 7(b)). As a result, the pretreatment of nigericin obviously deteriorates the benefits of melatonin on the modified Garcia score at 24 hours after MCAO (Figure 7(c), *p* < 0.01 vs. MCAO+DX+MEL).

## 4. Discussion

**Figure 8:**
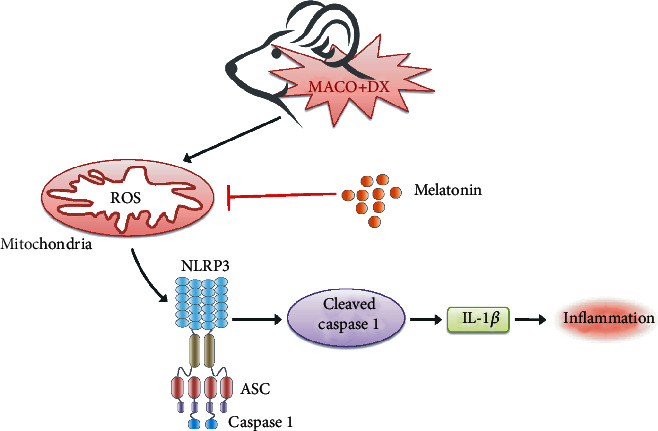
The pathway model of NLRP3 inflammasome-mediated inflammation. Under hyperglycemic cellular condition, large amounts of ROS are produced in mitochondria and accumulated intracellularly. This high concentration of ROS leads to the release of TXNIP from oxidized TRX and in turn directly binds to the leucine-rich region of NLRP3 (omitted in this figure), Subsequently, NLRP3 oligomerizes and recruits ASC and procaspase 1 to form the NLRP3 inflammasome complex, which triggers the activation of caspase 1 and the release of proinflammatory cytokines like IL-1*β*. Finally, inflammatory response is induced by abundant proinflammatory cytokines. DX: dextrose; ROS: reactive oxygen species; TXNIP: thioredoxin-interacting protein; TRX: thioredoxin; ASC: apoptosis-associated speck-like protein containing a caspase recruitment domain; IL-1*β*: interleukin-1*β*.

In the present study, we answer two questions: first, melatonin has the function to attenuate hyperglycemia-enhanced HT. Second, NLRP3 plays an important role in hyperglycemia-enhanced HT and melatonin attenuates HT through the ROS/TXNIP/NLRP3 pathway (Figure 8).

Both in clinic trials and in animal models, hperglycemia not only is the risk factor of stroke but also can induce the secondary brain injury after stroke such as HT [35–38]. It has been reported that several factors like matrix metalloproteinase, mitochondrial defects, and accumulation of ROS participate in hyperglycemia-enhanced HT after ischemic stroke in MCAO rats [35, 39–41]. ROS including oxide anion, hydrogen peroxide, and hydroxyl radical are the by-products mainly produced in mitochondria and endoplasmic reticulum where glucose and lipids were excessively oxidized. Interestingly, hyperglycemia is highly associated with the accumulation of these glucose and lipids [42]. Consistent with the theoretical, we observed the elevated ROS and increased modified Garcia score in the MCAO+DX group compared with the MCAO group. Our results indicated hyperglycemia can enhance oxidative stress and aggravate the neurological function in hyperglycemia-enhanced HT.

Melatonin (N-acetyl-5-methoxytryptamine) is an amine hormone primarily produced by the pineal gland of mammals and humans, the content of which is changed following a circadian rhythm. [43]. Initial researches reported the key role of melatonin in the maintenance of cell homeostasis and regulation of circadian rhythms [44, 45]. However, this hormone was also found to be involved in antioxidation [46, 47]. As a powerful antioxidant agent, melatonin has the function to reduce the concentration of ROS [7, 8] and therefore can be used to attenuates many diseases, such as Alzheimer's disease and traumatic brain injury [7, 48, 49]. Additionally, in animal models of ischemic stroke, melatonin has been shown to reduce the level of oxidative stress and regulate the change of antioxidant enzyme components to control values [50], which lead to the reduction of neuroinflammation induced by ROS accumulation [51]. Meanwhile, the inhibitory effect of melatonin on neuroinflammation can also be achieved through the transformation of microglia phenotype by regulating signal transducer and activator of transcription 3 (STAT3) in both *vivo* and *vitro* model [52]. However, no previous reports address the role of melatonin in hyperglycemia-enhanced HT. Previous studies reported that 15 mg/kg melatonin showed significant neuroprotection in both diabetic and hyperglycemic ICH rat models [53–55]. However, low dose of melatonin (5 mg/kg) failed to show effect on ameliorating in the ICH+DX model with more severe brain edema and neuron apoptosis [54]. In the present study, at the treatment of three doses of melatonin from low to high, we found that each dose can attenuate the outcome of hyperglycemia-enhanced HT, and the high dose had the best effect. Abundant previous studies have confirmed that melatonin possesses the function of antioxidant from low dose to high dose [56, 57]. Interestingly, the levels of NLRP3, cleaved caspase 1, and IL-1*β* were also found to be reduced when compared with the MCAO+DX group. Melatonin has been reported to inhibit the activation of NLRP3 inflammasome in various diseases including atherosclerosis, sepsis, and also CNS diseases [15, 58–60]. Surprisingly in CNS diseases, two successive studies showed that melatonin alleviated early brain injury induced by SAH via inhibiting NLRP3 inflammasome [15, 61]. Neurological benefit of NLRP3 inflammasome attenuation by melatonin was also found in LPS-induced acute depressive-like behaviors. More importantly, NLRP3 inflammasome was mainly expressed in microglia, which is consistent with our immunofluorescence results [58].

After confirming the melatonin's positive effects on hyperglycemia-enhanced HT, we further explored the mechanism underlying melatonin-mediated neuroprotective effects. Hung et al. reported that melatonin inhibits the activation and expression of matrix metalloproteinase-9 to attenuate transient focal cerebral ischemia followed by reperfusion-induced hemorrhage in rats [10]. Inflammation may play a critical role in the pathophysiology of hyperglycemia-enhanced HT. Our previous work in the same hyperglycemic rats after MCAO revealed that inflammation and blood-brain barrier disruption were aggravated by the ROS/TXNIP/NLRP3 signaling pathway, which can be attenuated by the hyperbaric oxygen precondition [28]. In this present study, we focus on the NLRP3 inflammasome, which was originally regarded as the key component in innate immune system, including NLRP3 and ASC as well as the cysteine protease caspase-1. This inflammasome have been reported to have a detrimental effect in the ischemic stroke and intracerebral hemorrhage model [15–17, 61] . However, there is no report about the role of NLRP3 inflammasome in hyperglycemia-enhanced HT. NLRP3 inflammasome can be induced by pathogen-associated and damage-associated molecular patterns including particulate factors like basic calcium phosphate crystals and cholesterol crystals as well as soluble factors like extracellular ATP and nigericin [26]. Here, we try to explore the potential mechanism of melatonin protection by introducing nigericin to activate NLRP3 inflammasome. As we can see, in the MCAO+DX group, the concentration of ROS was exceptionally high. At this situation, TXNIP is released from oxidized TRX secondary to high concentrations of ROS and in turn directly binds to a specific region of NLRP3 with abundant leucine which leads to assembly and activation of inflammasome [19]. Consistent with the theory, in the present study, we found that the components of NLRP3 inflammasome, NLRP3 and cleaved caspase-1, were significantly increased in the MCAO+DX group and the modified Garcia score was significantly lower in the MCAO+DX group compared with the MCAO group. Accordingly, the downstream of NLRP3 inflammasome, such as IL-1*β* and TNF, was all increased in the MCAO+DX group. These inflammatory cytokines can damage the tight junction of endothelial cells, then damage the integrity of the blood-brain barrier, which results in the aggravated of HT [28]. Fortunately, melatonin can attenuate the detrimental effect caused by NLRP3 inflammasome. In the MCAO+DX+melatonin group, the expression of NLRP3, caspase-1, and the downstream inflammatory cytokines IL-1*β* was significantly decreased; simultaneously, the neurological function was significantly improved. More importantly, the hemorrhage volume was attenuated. All of these neurological benefits can be significantly reversed by the administration of nigericin. Especially, downstream protein represented by cleaved caspase 1 and IL-1*β* changed simultaneously with NLRP3. Regretfully, we did not verify the function and expression change of ROS and TXNIP upstream NLRP3 after the injection of nigericin in this present work. Nevertheless, Tschopp and Schroder reviewed that all NLRP3 agonists trigger the association of NLRP3 with TXNIP in a ROS-dependent manner [14] and more recent melatonin treatment studies [62–64] seem to reveal the ROS/TXNIP/NLRP3 axis as one of fixed pathways. Moreover, this signaling pathway in the same hyperglycemic MCAO rat model has been rigorously verified in our previous work. So, there are grounds to speculate that ROS/TXNIP/NLRP3 axis is a potential pathway mediating neuroprotection of melatonin. Additionally, it is worth noting that the effect of melatonin on hyperglycemia-enhanced HT was similar with the ROS scavenger conducted in our previous work [28]. The good news is that melatonin has few side effects.

HT has severe influence on early mortality and prognosis of ischemic stroke. To better simulate clinical diabetes, in this study, we give administered DX before MCAO to artificially make a condition of hyperglycemia. We demonstrated the important role of NLRP3 inflammasome in the process of hyperglycemia-enhanced HT and melatonin's ability to suppress the expression of the NLRP3 inflammasome. Thus, melatonin may be a useful drug to prevent HT in diabetic, and NLRP3 inflammasome may become a new therapeutic target in hyperglycemia-enhanced HT. However, there are some limitations. Firstly, ROS can induce several inflammatory pathways, ROS/TXNIP/NLRP3 being one of them. Additionally, other potential pathways with efficient advantage on neurobehavioral functions still cannot be excluded.

## 5. Conclusion

In conclusion, melatonin has the function to attenuate hyperglycemia-enhanced HT. NLRP3 plays an important role in hyperglycemia-enhanced HT, and melatonin attenuates HT through the ROS/TXNIP/NLRP3 pathway.

## Data Availability

The data used to support the findings of this study are available from the corresponding author upon request.

## Abbreviations

MCAO: Middle cerebral artery occlusion
DX: Dextrose
ROS: Reactive oxygen species
NLRP3: Nod-like receptor protein 3
HT: Hemorrhagic transformation
ASC: Apoptosis-associated speck-like protein containing a caspase recruitment domain
TXNIP: Thioredoxin-interacting protein
TRX: Thioredoxin.
